# Exogenous Attention to Emotional Stimuli Presenting Realistic (3D) Looming Motion

**DOI:** 10.1007/s10548-022-00909-w

**Published:** 2022-08-06

**Authors:** Uxía Fernández-Folgueiras, María Hernández-Lorca, Constantino Méndez-Bértolo, Fátima Álvarez, Tamara Giménez-Fernández, Luis Carretié

**Affiliations:** 1 Departamento de Psicología Biológica y de la Salud, Facultad de Psicología, Universidad Autónoma de Madrid, Calle Ivan Pavlov 6, 28049 Madrid, Spain; 2 Departamento de Psicología. Facultad de Ciencias de la Educación, Universidad de Cádiz, Calle de la República Saharaui 12, 11519 Cádiz, Spain; 3 Instituto de Investigación Biomédica de Cádiz (INIBICA), Avenida Ana de Viya 21, 11009 Cádiz, Spain; 4 Departamento de Psicología Básica, Facultad de Psicología, Universidad Autónoma de Madrid, Calle Ivan Pavlov 6, 28049 Madrid, Spain

**Keywords:** Exogenous attention, Looming motion, Emotion, ERPs, 3D

## Abstract

Previous research shows that dynamic stimuli, on the one hand, and emotional stimuli, on the other, capture exogenous attention due to their biological relevance. Through neural (ERPs) and behavioral measures (reaction times and errors), the present study explored the combined effect of looming motion and emotional content on attentional capture. To this end, 3D-recreated static and dynamic animals assessed as emotional (positive or negative) or neutral were presented as distractors while 71 volunteers performed a line orientation task. We observed a two-phase effect: firstly (before 300 ms), early components of ERPs (P1p and N2po) showed enhanced exogenous attentional capture by looming positive distractors and static threatening animals. Thereafter, dynamic and static threatening distractors received enhanced endogenous attention as revealed by both late ERP activity (LPC) and behavioral (errors) responses. These effects are likely explained by both the emotional valence and the distance of the stimulus at each moment.

## Introduction

Our environment is dynamic and unpredictable, full of moving stimuli with which we may interact. Throughout evolution, we have developed a default priority system to detect dynamic events as they could be potentially harmful. Therefore, detecting and responding adequately to them is critical for an adjusted interaction with the environment (crossing the road or driving, for example). The automatic detection (also named exogenous or bottom-up attention) of dynamic events seems crucial for this interaction, as it interrupts the endogenous attention to the current target and allows attentional reorientation towards salient or biologically relevant stimuli (Carretié 2014; Corbetta et al. 2008; Corbetta and Shulman 2002; Posner and Petersen 1990).

Behavioral research in human adults confirms that our visual system prioritizes moving events that require a behaviorally urgent response (Lin et al. 2008). Franconeri and Simons (2003) proposed the behavioral-urgency hypothesis suggesting that certain types of motion capture attention because they may indicate potential threat or collision. This would be the case for looming or approaching motion, for example. Receding motion, however, does not signal a potential risk, leading to conclude that not all dynamic events may capture attention (Franconeri and Simons 2003, 2005; Lin et al. 2008). Several studies also supported this hypothesis, which confirms the attentional advantage of looming motion. Von Mühlenen and Lleras (2007) found, through a probe-detection task, that looming motion receives attentional priority compared to any other type of motion as up-, down-, left-, rightwards, and also receding or motion onsets. Skarrat, Cole, and Gellatly (2009) showed that both looming and receding motion received prioritized attention compared to static stimuli, but reaction times were faster in looming motion trials in a visual search task. These authors suggested that the enhanced processing of looming motion is beyond the effects of motion alone. In this line, Parker and Alais (2007) presented simultaneously looming and receding stimuli (expanding and contracting stimuli) using a binocular rivalry paradigm and found that looming was the dominant image compared to receding. Hence, there is considerable evidence that looming stimuli are prioritized probably due to an alerting effect that requires immediate action (Ono and Kitazawa 2010; Rossini 2014), and this effect appears to influence automatically, independent of conscious awareness (Hervais-Adelman et al. 2015; Judd et al. 2004; Lin et al. 2009).

Besides behavioral data, event-related potentials (ERPs) may provide relevant information on the mechanisms underlying exogenous attention to dynamic events since they may disentangle rapid attention-related neural processes that cannot be distinguished behaviorally. Scarce data in this respect also point to an exogenous attention bias towards dynamic stimuli. The majority of ERP studies showing exogenous attention to motion traditionally employed moving sinusoidal gratings in the periphery of the visual field while the participants performed a visual central task (Amenedo et al. 2007; Pazo-Álvarez et al. 2004a, b). These peripheral distractors were presented following an oddball paradigm so that they moved upwards (standard stimuli) or downwards (deviant or infrequent stimuli). The main results showed that all dynamic stimuli elicited a P1-N2-P2 complex, but infrequent motion direction also elicited enhanced N2 amplitudes at posterior scalp locations, confirming that visual motion is processed pre-attentively. However, and to our knowledge, no ERP research has explored the neural mechanisms underlying exogenous attention to looming stimuli.

In evolutionary terms, emotion is a feature prone to capture attention. Threatening or appetitive stimuli, which are emotional by definition, are preferentially processed among other events relevant for survival. Exogenous attention to emotional stimuli is often explored through *concurrent but distinct target-distractor* (CDTD) tasks, also named directed attention tasks (MacNamara et al. 2012). This experimental paradigm consists of the simultaneous presentation of targets (i.e., elements to which participants are asked to direct their endogenous attention) and distractors (i.e., emotional stimuli irrelevant to the task), but the former and the latter are physically segregated (Carretié 2014). Most studies employing CDTD tasks find some behavioral and/or neural exogenous attention bias toward emotional (positive and negative) distractors compared to neutral. At the behavioral level, attentional capture by emotional distractors produces disruption in the ongoing task consisting of increased reaction times and/or error rates in response to targets (see a review in Carretié 2014; and later works by Carboni et al. 2017; Tiferet-Dweck et al. 2016). Neural (ERP) indices of exogenous attention in CDTD tasks also show that emotional distractors preferentially capture it compared to neutral, and this modulation is mainly observed at early latencies (within the first 300 ms after stimulus onset). These effects are manifested as enhanced amplitudes in response to emotional (both positive and negative) when compared to neutral stimuli at the posterior P1 (P1p; Mendoza-Medialdea & Ruiz-Padial 2021; Soares et al. 2017), anterior P2 (P2a; Carretié and Ruiz-Padial 2016), and the family of N2 components distributed along different scalp sites (N2x; Carretié et al. 2017; Ruiz-Padial & Mercado, 2021).

Taken together, these studies showed that both motion and emotion separately grab attention to a greater extent than static and non-emotional stimuli, respectively. However, research combining both variables is surprisingly scarce, considering our real environment is full of dynamic emotional events. In the field of face processing, Martin et al., (2021, experiment 1) recently examined the effect of attentional capture by emotional faces presented in either looming or receding conditions. Participants were instructed to focus their attention on a central task while ignoring the looming or receding fearful and neutral faces presented bilaterally. Although emotional faces or their interaction with motion did not interfere with performance, greater amplitudes of lateralized N170 (a face-sensitive ERP component) and N2pc components of the ERPs were found for the looming upright fearful in comparison to non-emotional or inverted looming faces. Authors claimed that stimuli that combined the threatening and approaching characteristics enhanced attention at the early processing stages. Convergently, Carretié et al., (2009) examined whether negative (spiders and cockroaches) or non-negative (butterflies or ladybirds) distractors presented in the periphery, either dynamic or static, captured attention in a CDTD task in which participants performed a central task. These authors reported that negative distractors caused worse performance in the task (higher reaction times and error rates) and greater P1p amplitudes. However, the motion explored in this study was from the periphery towards the fixation point and not looming.

The present study explored the attentional capture by looming emotional (positive and threatening) and non-emotional stimuli, both behaviorally and through ERPs. Participants performed a CDTD task where distractors consisted of static or looming animals. Animals are a proper choice since they may remain static or dynamic in natural circumstances, and some of them are commonly perceived as threatening (e.g., snakes or spiders) and others as non-threatening, including neutral (e.g., fishes or birds) or positive (e.g., rabbits or puppies), as suggested by normative ratings (e.g., EmoMadrid: Carretié et al. 2019; IAPS: Lang et al. 2005). Targets consisted of two lines presented on the screen, and participants were asked to indicate whether both lines had the same orientation (were concordant) or not (were discordant). In addition to the looming motion (relative to static) and the inclusion of positive stimuli along with negative and neutral (positive stimuli are much less employed than negative), our research intends to increase perceptual realism. Concretely, it includes a 3D motion recreation methodology. All the studies focused on motion reviewed above have employed bidimensional (2D) apparent motion by increasing or decreasing the stimulus size in looming and receding stimuli, respectively. Here, we employ shutter glasses that, in congruence with a high-resolution LCD monitor, generate a stereoscopic vision and lead the participant to perceive the image in 3D. The use of 3D stimulation provides more realistic looming motion, increasing the ecological validity of this study, aiming to reflect the cognitive mechanisms naturally involved in motion processing. We hypothesize that looming emotional (especially threatening) stimuli would elicit increased attentional capture, resulting in a disruption in the ongoing task by emotional distractors (higher error rates and/or reaction times) and enhanced amplitudes in ERP components indexing exogenous attention such as P1p, P2a and/or N2x.

## Method

### Participants

Seventy-seven volunteers participated in the study, although data from only 71 of them were analyzed, as explained later (57 women; age range = 18–31, mean = 19.64 years, *SD* = 2.14 years). This sample size gave us an optimal statistical power (1− β = 0.80) even to reliably detect small effects (η^2^_p_ = 0.10) for the more restrictive main effect involving the two-level factor (Motion) in a 2 × 3 ANOVA design. All power calculations were computed using the MorePower 6.0.4 calculator (Campbell and Thompson 2012). The study was previously approved by the Universidad Autónoma de Madrid's ethics committee. All participants were students of Psychology, provided their informed consent according to the Declaration of Helsinki, and received academic compensation for their participation.

### Stimuli and Procedure

**Fig. 1:**
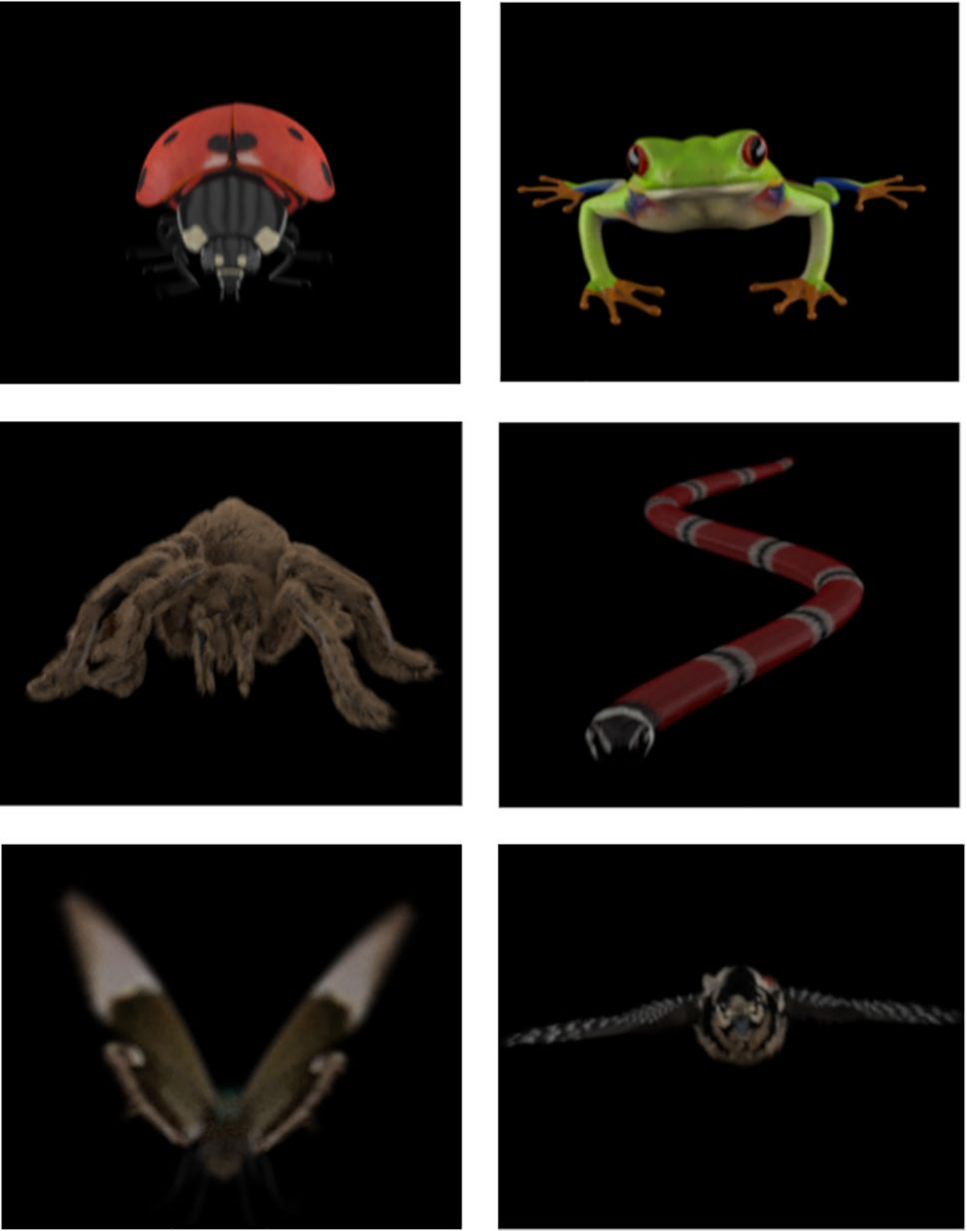
Examples of static distractors used in the experiment. The two upper images represent the positive distractors (ladybird and frog), the middle images the negative (spider and snake), and the two bottom images ones the neutral (butterfly and bird)

**Fig. 2:**
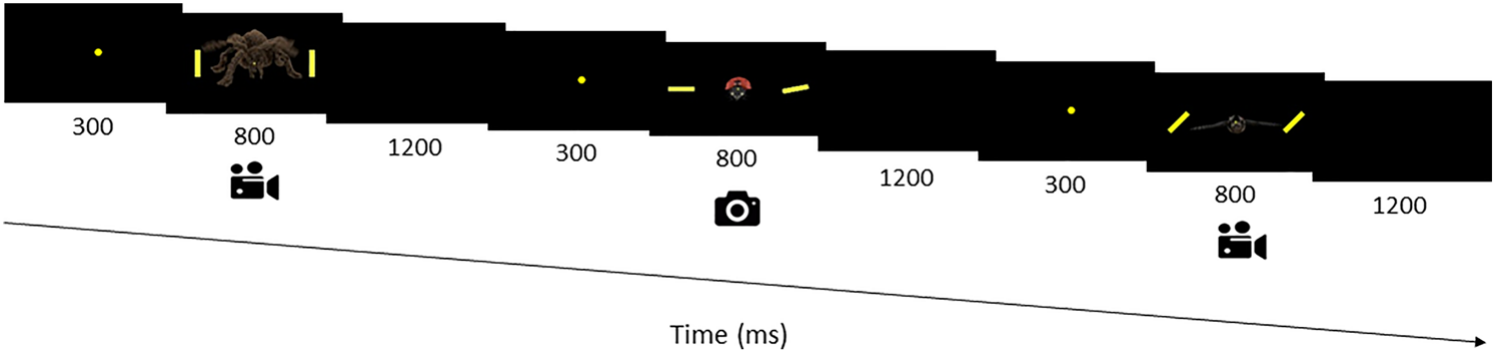
Schematic illustration of the CDTD task with three trials. The distractors consisted of emotional animals (from left to right: threatening, positive, and neutral), which might appear static or approaching the participants. All trials contained two lines (target) on both sides of the emotional distractor, to which participants had to respond

Participants were placed in an electrically shielded, sound-attenuated room at 100 cm from the stimulation screen. Stimuli were presented on a ViewPixx© screen using Psychtoolbox 3 task programming extensions for MatLab (Kleiner et al. 2007). Participants were instructed to perform a line orientation (CDTD) task in which they were required to press “as accurately and rapidly as possible” one key if both lines (target) presented to the left and the right of the fixation point had the same orientation (i.e., had concordant orientation), and a different key if both lines had different orientation with respect to each other (i.e., discordant orientation). Simultaneously, distractors (looming and static animals) were centrally presented: Figs. 1 and 2. All these elements appeared over a plain black background. To increase ecological validity, all stimuli were presented in 3D on the screen through a 3DPixx LCD with shutter glasses that occluded one eye at a time, 60 times/second per eye, in congruence with the refresh rate of the monitor (120 Hz). This setting led the participant to perceive a different image in each eye and recreate a three-dimensional space (in front of and behind the actual screen plane). An initial test was carried out to ensure the capability of 3D perception through the LCD glasses in our participants. In this test, we presented a picture different from those employed in the experimental run. All participants achieved 3D vision through the LCD glasses in this test previous to the task.

Target lines (3.15° × 0.57° wide each) were presented in the periphery at both sides of the screen (9.8° from their center to the middle of the fixation dot). We established 32 line combinations according to their inclination angle: half of them presented the same orientation (16 concordant combinations), and the other half had different orientations (16 discordant combinations). In concordant trials, both lines presented the same slope (the rotation angles were: 11.25°, 22.50°, 33.75°, 45°, 56.25°, 67.50°, 78.75°, 90°, 101.25°, 112.50°, 123.75°, 135°, 146.25°, 157.50°, 168.75°, 180°) whereas discordant presented a difference of 11.25° between both lines. The key-response assignment was counterbalanced across participants to indicate whether both lines were concordant or discordant. Importantly, targets were identical (and their ERP effects homogeneous) across conditions in terms of physical characteristics: size, color, location, and orientation.

Distractors consisted of computer-generated pictures developed by an expert in graphic design. Dynamic and static stimuli –looming motion clips or a single photogram, respectively-were emotionally loaded animals. Two exemplars per emotional category were presented (six animals in total): threatening (a spider or a snake), positive (a ladybird or a frog), or neutral animals (a bird or a butterfly). Each exemplar was repeated 64 times − 32 dynamic and 32 static-, resulting in 384 total trials. All these stimuli were selected from a broader set of animals as a function of their Valence and Arousal rating provided by an independent sample of 94 healthy volunteer students from the Universidad Autónoma de Madrid (Table 1). Hence, six conditions were implemented: threatening dynamic (dNeg) and static (sNeg), positive dynamic (dPos) and static (sPos), and neutral dynamic (dNeu) and static (sNeu). Figure 1 shows the static version of each distractor presented to participants, and both dynamic and static versions can be visualized -monocular version- at https://osf.io/5p94k/. Although the trajectory of the moving animals started and ended at the same location, the idiosyncratic motion pattern of each animal (which depends on whether it moves by walking, flying, or jumping, since looming motion tried to reproduce the typical motion characteristics of each species) results in slightly different looming motions: continuous linear in the case of spider, ladybird, and bird, smooth zigzag in the case of the snake and butterfly, and a discontinuous linear motion (that includes a jump) for the frog. In dynamic stimuli (always presenting a looming motion, as indicated), the area of the biggest animal in figure-ground terms (i.e., spider) oscillated between 3.44° × 5.72° (wide) in the "farthest" position and 6.30° × 9.72° in the "closest", and in the case of the smallest animal (ladybird), the area ranged between 1.72° × 2° and 3.44° × 3.15°. Size differences were not significant among conditions, neither the width nor the height of the images. These results were submitted to Kruskal–Wallis non-parametric tests since data did not achieve normality [Shapiro–Wilk test of normality: W(24) = 0.894, *p* = 0.016; Kruskal–Wallis test on the “farthest” position: Width χ^2^(2) = 1.143; *p* = 0.565, and Height χ^2^(2) = 4.191; *p* = 0.123, and in the “closest” position: Width χ^2^(2) = 2.000; *p* = 0.368, and Height χ^2^(2) = 3.603; *p* = 0.165]. The path of all animals was always rectilinear towards the viewer and crossed the fixation (foveal) point, a yellow dot (0.23° × 0.23°) in the center of the screen. The static stimuli were always centrally presented and consisted of the frame (or photogram) of each clip in which the corresponding animal was at the midpoint of their path. The area of the biggest animal in figure-ground terms (the spider, as indicated) in the static condition was 3.44° × 6.87°, and for the smallest animal (ladybird) was 2.29° × 2.29°.

**Table 1:** Means and standard deviation (in parenthesis) of Valence and Arousal ratings provided to each stimulus, and each experimental condition evaluated by an independent sample of ninety-four participants to select stimuli

	Negative			Positive			Neutral		
	Spider	Snake	Wasp	Butterfly	Frog	Ladybird	Ant	Bird	Fish
Valence (n = 94)	1.31 (0.64)	1.70 (0.90)	2.14 (0.99)	2.37 (0.97)	3.53 (0.93)	3.52 (1.08)	1.62 (0.85)	3.01 (0.95)	1.82 (0.83)
	1.72 (0.56)			3.14 (0.72)			2.15 (0.59)		
Arousal (n = 94)	4.49 (0.83)	4.29 (0.78)	3.92 (0.87)	3.34 (0.97)	3.21 (0.92)	2.65 (1.05)	3.97 (0.94)	3.41 (0.78)	3.82 (1.07)
	4.22 (0.63)			3.07 (0.68)			3.73 (0.62)		

Both dimensions range from 1 (very negative/relaxing) to 5 (very positive/arousing)

The whole set of 384 trials (64 per condition, as indicated: 32 concordant and 32 discordant) was displayed randomly in three blocks of 128 (approximately 5 min each), separated by a rest period of about 3 min. As shown in Fig. 2, stimuli (comprising targets and distractors) were presented for 800 ms, and the intertrial interval lasted 1200 ms. Along with the line orientation task instructions, participants were instructed to continuously fixate their gaze on the fixation dot and avoid blinking as much as possible during stimulus presentation. Before the experimental run, participants completed a practice block of 10 trials presenting static neutral objects. After completing the experimental run, all participants assessed the stimuli employed in the task in terms of Valence and Arousal on a Likert scale from 1 (very negative/very relaxing) to 5 (very positive/very arousing). The results of this affective evaluation were submitted to Friedman's non-parametric tests since data did not achieve normality [K-S tests: D(426) = 0.166 and 0.174 in Valence and Arousal dimensions, respectively, *p* < 0.001 in both cases]. As Table 2 shows, results followed the expected pattern: all conditions differed from each other in the Valence dimension showing a Pos > Neu > Neg pattern [Friedman’s test on Valence: χ^2^(2) = 86.125; *p* < 0.001, and pairwise Wilcoxon signed rank test between Pos vs. Neg: Z = − 6.919, *r* = 0.581; Pos vs. Neu: Z = − 5.281, *r* = 0.443, and for Neu vs. Neg: Z = − 5.785, *r* = 0.485, *p* < 0.001 in all cases], whereas in the Arousal dimension there were no differences between Neu and Pos but both differed from the Neg condition, rated as the most arousing (Neg > Pos/Neu) [Friedman’s test on Arousal: χ^2^(2) = 45.257; *p* < 0.001, and pairwise Wilcoxon signed rank test for Pos vs. Neu: Z = − 1.718, *p* = 0.086, *r* = 0.144; Pos vs. Neg: Z = − 5.351, *r* = 0.449, and for Neu vs. Neg: Z = − 4.693, *r* = 0.394, *p* < 0.001 for both significant comparisons] (see Table 2). To prevent potential perceptual biases, we measured the luminosity means and the intensity of red, green, and blue (RGB) channels of each picture with Adobe Photoshop© CC 2015. These measures were homogeneous in luminosity and intensity of RGB for the picture set, as the maximal difference between emotional categories was 0.48% of the total luminance range (defined here as the luminance emitted by a white screen minus the luminance emitted by a black screen) and < 1% of total range (from 0 to 255) intensity of RGB channels (red: 0.8%, green: 0.45%, and blue: 0.35%): see Table 2.

**Table 2:** Means and standard deviation (in parenthesis) of luminosity and intensity of red, green, and blue channels (0–255), in addition to Valence and Arousal dimensions of Threatening, Positive and Neutral stimuli (1 = very negative/very relaxing to 5 = very positive/very arousing) evaluated by the experimental sample of seventy-one participants after completing the task

	Negative		Positive		Neutral	
	Spider	Snake	Ladybird	Frog	Bird	Butterfly
Luminosity	4.69 (16)	1.32 (8.06)	1.8 (12)	3.61 (19.2)	1.2 (7.85)	2.36 (7.12)
	3.00 (12.03)		2.70 (15.6)		1.78 (7.48)	
Red	5.63 (19.2)	2.05 (12.29)	2.56 (18.68)	3.84 (20.48)	1.28 (8.70)	2.30 (13.31)
	3.84 (15.74)		3.2 (19.58)		1.79 (11.00)	
Green	4.61 (15.36)	1.02 (6.91)	1.54 (9.984)	3.84 (20.99)	1.28 (7.94)	2.05 (11.52)
	2.82 (11.14)		2.69 (15.49)		1.66 (9.73)	
Blue	3.33 (12.03)	1.02 (6.65)	1.28 (8.70)	1.79 (10.24)	1.02 (7.17)	1.54 (9.98)
	2.18 (9.34)		1.54 (9.47)		1.28 (8.58)	
Valence (n = 71)	1.62 (0.99)	2.08 (1.01)	3.60 (0.95)	3.45 (0.86)	2.93 (0.95)	2.80 (1.07)
	1.85 (0.83)		3.53 (0.73)		2.87 (0.78)	
Arousal (n = 71)	3.75 (1.45)	3.97 (1.04)	2.72 (1.02)	3.04 (1.01)	3.07 (0.93)	3.14 (0.95)
	3.86 (0.99)		2.88 (0.78)		3.10 (0.66)	

### Recording and Preprocessing

Electroencephalographic (EEG) activity was recorded using an electrode cap (ElectroCap International) with tin electrodes. Fifty-nine electrodes were placed at the scalp following a homogeneous distribution and the international 10–20 System. All scalp electrodes were referenced to the nose tip. Electrooculographic (EOG) data were recorded supra- and infra-orbitally (vertical EOG) as well as from the left versus right orbital rim (horizontal EOG). An online analog bandpass filter of 0.3 Hz to 10 kHz was applied. Recordings were continuously digitized at a sampling rate of 420 Hz. An offline digital bandpass filter of 0.3 to 30 Hz was applied using the Fieldtrip software (https://www.fieldtriptoolbox.org; Oostenveld et al. 2011). The continuous recording was divided into 1000 ms epochs for each trial, beginning 200 ms before stimulus onset. Behavioral responses were recorded through a numeric keypad. Outlier trials (with responses before 250 ms or after 2000 ms) were eliminated.

Ocular artifact removal was carried out through an Independent Component Analysis based strategy (Jung et al. 2000) as implemented in Fieldtrip. After this process, the second stage of visual inspection of EEG data was conducted. If any further artifact was present, the corresponding trial was discarded. This procedure led to the average admission of 58.55 (SD = 4.43) dNeg trials, 57.66 (5.04) sNeg, 58.07 (4.80) dPos, 58.11 (4.71) sPos, 58.20 (4.85) dNeu and 58.37 (4.91) sNeu, with the difference among conditions being non-significant [K-S test: D (426) = 0.145, *p* < 0.001. Friedman’s test: χ^2^(5) = 4.664, *p* = 0.458] (see Table 3). The minimum number of trials accepted for averaging was 38 trials per participant and condition. Six participants were discarded from the analyses: four of them due to an excessive number of interferences (noisy channels were recovered through interpolation from neighbor electrodes to a maximum of 10% of the total number of electrodes, a limit surpassed in these four cases), and two participants due to technical failures in the presentation of stimuli. In addition to these correction and rejection strategies, we carried out additional analyses to discard any significant horizontal ocular activity in the results since the lines they had to pay attention to were presented on both sides of the fixation dot (Fig. 2). Thus, repeated-measure analyses of variance (ANOVAs) introducing Motion (dynamic, static) and Emotion (threatening, positive, and neutral) as factors were performed on horizontal EOG (hEOG) data as control analyses (see Results section).

**Table 3:** Means and standard deviations (in parenthesis) of (i) average number of trials, (ii) error rates, and (iii) reaction times

	dNeg	sNeg	dPos		sPos	dNeu	sNeu
Trials	58.55 (4.43)	57.66 (5.04)	58.07 (4.80)		58.11 (4.71)	58.20 (4.85)	58.37 (4.91)
Average number of trials							
Behavior							
Errors	11.60 (3.52)	11.74 (3.41)	23.14 (5.01)	22.28 (5.43)		22.91 (4.96)	22.79 (4.74)
Reaction times (ms)	800 (17.6)	792 (17.1)	796 (17.1)	803 (16.7)		795 (16.6)	800 (16.7)

### Data Analysis

#### Detection, Spatiotemporal Characterization, and Quantification of Relevant ERP Components

To detect and quantify relevant components (P1p, P2a, and N2x), we employed a two-step Principal Component Analysis (PCA), a strategy that has repeatedly been recommended for data reduction purposes in order to reliably define individual ERP components and to manage component overlap (Chapman et al. 2004; Dien 2010). Briefly, temporal PCA (tPCA) computes the covariance between all ERP time points, which tends to be high between those involved in the same component and low between those belonging to different components. The solution is a set of factors composed of highly covarying time points, which ideally correspond to ERP components. Extracted temporal factors (TF) are quantified as factor scores, linearly related to amplitudes (Dien 2010, 2012). The decision on the number of factors to select was based on the scree test (Maxwell and Cliff 2006). Extracted factors were submitted to promax rotation, as previously recommended (Dien 2010). Once quantified in temporal terms and prior to statistical contrasts, relevant temporal components were decomposed into their main spatial regions via a spatial PCA (sPCA). Thus, while tPCA separates ERP components over time, sPCA separates them in space, each scalp region or spatial factor (SF) ideally reflecting one of the concurrent neural processes underlying each TF. This spatial decomposition is an advisable strategy prior to statistical contrasts, given that ERP components may behave differently in some scalp areas than in others. Basically, each SF is formed with the scalp points where recordings tend to covary. As in the case of tPCA, the decision on the number of spatial factors to select was based on the scree test, and extracted factors were submitted to promax rotation as well. Statistical analyses were computed on spatial factor scores, which are linearly related to amplitudes.

#### Analyses on Experimental Effects

Concerning behavior, average reaction times (in milliseconds) and number of errors (defined here as average of incorrect and blank responses) for each participant in each condition were submitted to non-parametric contrasts (Wilcoxon signed-rank test), due to their non-Gaussian distribution [K-S test on reaction times: D (426) = 0.158, *p* < 0.001; on number of errors: D (426) = 0.070, *p* < 0.001]. Effect sizes for these non-parametrical tests were computed using the procedure described by Pallant (2007, pp. 224–225). Means and standard deviations of behavioral data are presented in Table 3.

With respect to ERPs, two-way repeated-measures 2 × 3 ANOVAs on spatial factor scores were carried out on Motion (dynamic, static) and Emotion (threatening, positive, neutral) as within-participant factors. The interaction of Motion x Emotion was analyzed only in those components sensitive to any of the main effects (either Motion or Emotion). We used the Greenhouse–Geisser (G-G) epsilon correction to adjust the degrees of freedom of the F ratios if necessary, and post-hoc comparisons were performed to determine the significance of pairwise contrasts using the Bonferroni correction procedure. Effect sizes were computed using the partial eta-square (η^2^_p_) method. The analyses were carried out using SPSS 20.0 software package (IBM SPSS Inc., 2011).

## Results

### Experimental Effects on Behavior

**Fig. 3:**
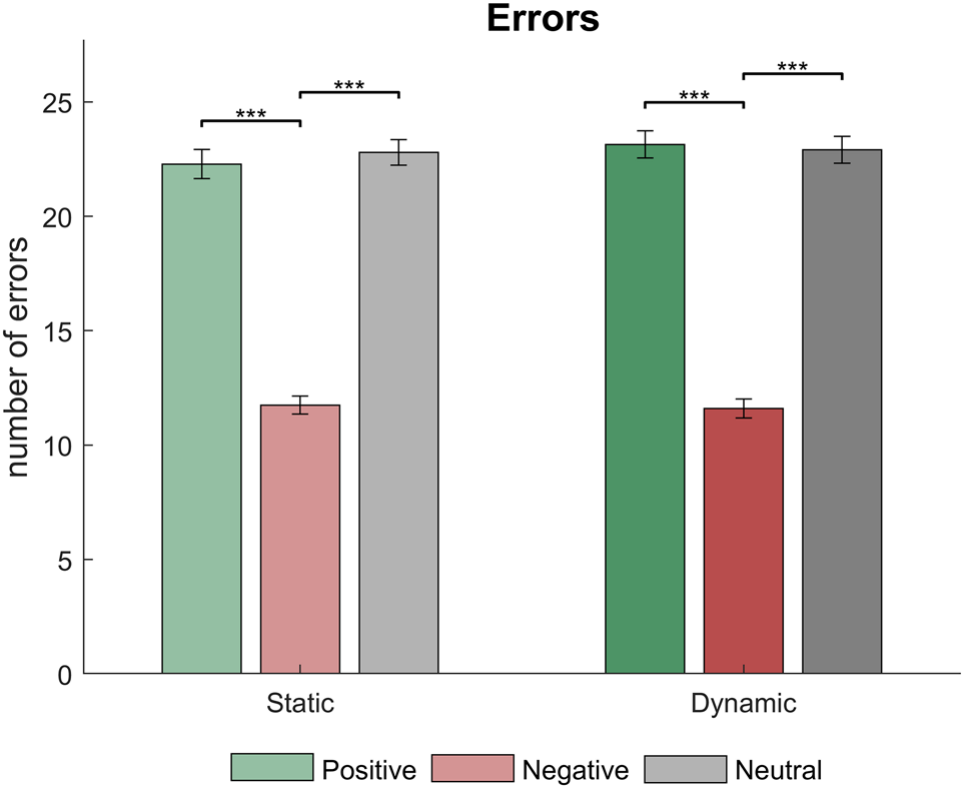
Bar graphs representing behavioral data (error rates) for each condition

Results on number of errors yielded a main effect of Emotion [Friedman’s test: χ^2^(2) = 211.468, *p* < 0.001]. The post-hoc pairwise test indicated that Neg trials elicited lower number of errors than Pos and Neu [Z = − 10.338, *r* = 0.867 and Z = − 10.307, *r* = 0.865, both *ps* < 0.001). Non-significant differences were found between Pos and Neu conditions [Z = − 0.461, *p* = 0.645, *r* = 0.039]. We did not find a main effect of Motion [χ^2^(1) = 0.005, *p* = 0.943]. Regarding the Emotion x Motion interaction, Wilcoxon signed-rank test showed that Neg stimuli elicited lower number of errors than Pos and Neu in both dynamic [dPos > dNeg: Z = − 7.328, *r* = 0.615; dNeu > dNeg: Z = − 7.276, *r* = 0.611; both significant *ps* < 0.001], and static conditions [sPos > sNeg: Z = − 7.315, *r* = 0.614; sNeu > sNeg: Z = − 7.327, *r* = 0.615, both significant *p* < 0.001]. Results involving Pos and Neu comparisons were non-significant [dPos vs dNeu: *p* = 0.760; sPos vs sNeu: *p* = 0.334] (see Fig. 3). Regarding reaction times, results revealed non-significant main effects of Emotion [Friedman’s test: χ^2^(2) = 2.662, p = 0.264] nor Motion [χ^2^(1) = 2.934, p = 0.087], so this behavioral parameter was not further explored.

### ERP Data: P1p, P2a, N2x, and LPC

**Fig. 4:**
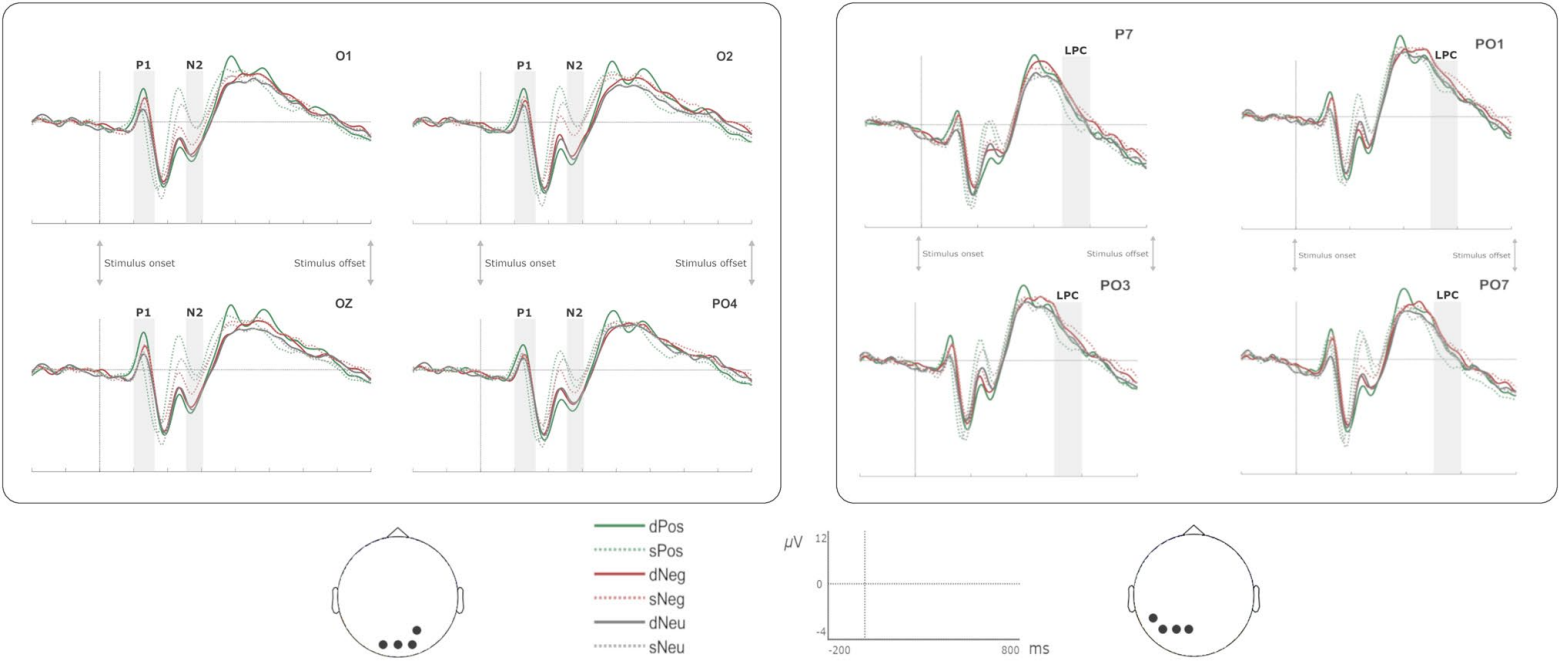
Grand averages of the ERP components. The left side of the figure represents grand averages at parieto-occipital scalp locations (O1, O2, Oz, and PO4) for each component indexing exogenous attention (P1p and N2po) in response to static and dynamic stimuli (dNeg, sNeg, dPos, sPos, dNeu, and sNeu). The right side represents grand averages of channels showing maximum loadings in left occipital (P7, PO1, PO3, PO7) electrodes for endogenous attention (LPC) attention in response to static and dynamic stimuli (dNeg, sNeg, dPos, sPos, dNeu, and sNeu)

**Fig. 5:**
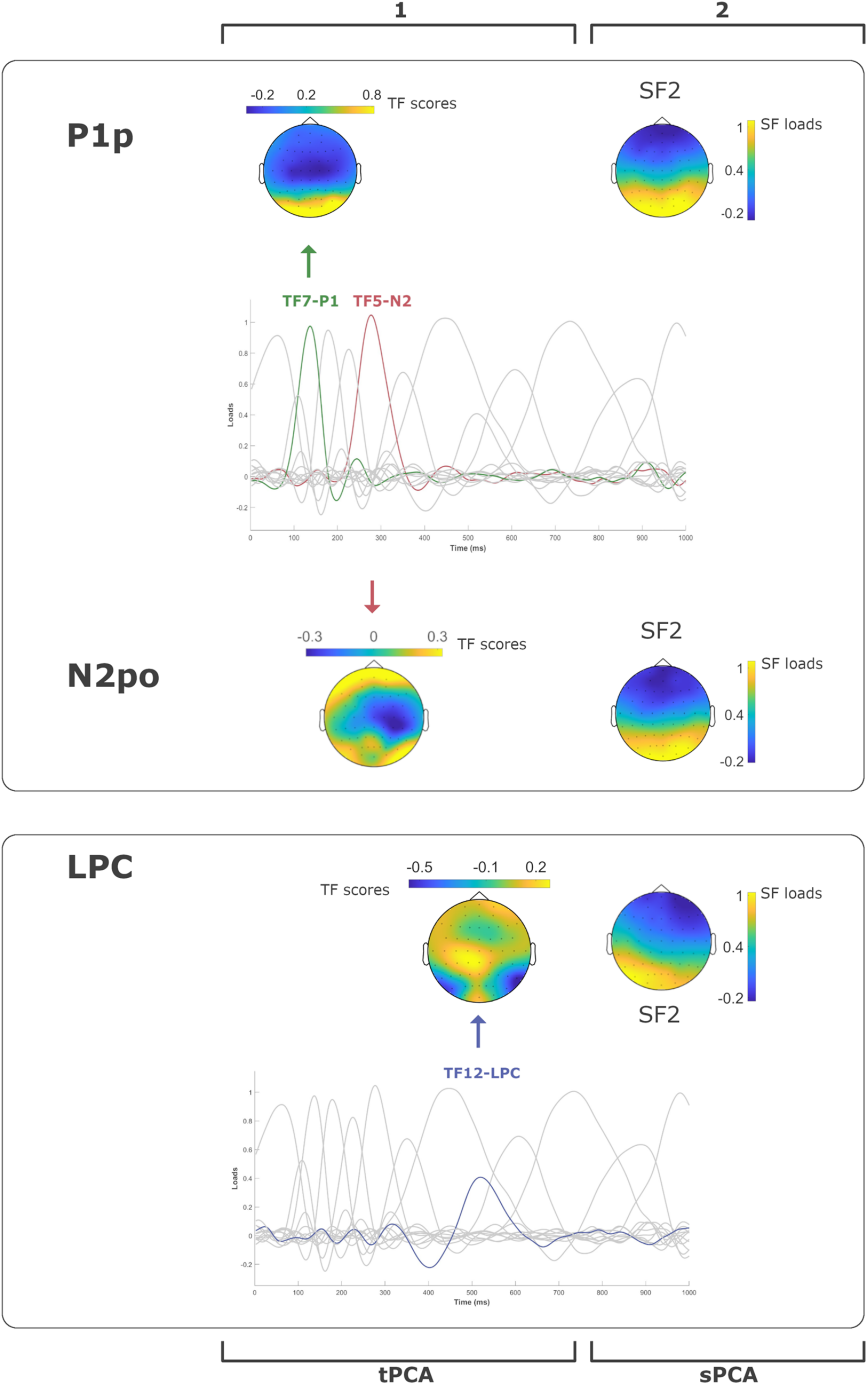
Two-step principal component analysis (PCA) analysis structure. First, the temporal PCA (tPCA) extracted temporal factors (TF) or components from original recordings, being P1p, N2po (TF7 and TF5, respectively), and LPC (TF12), those relevant to our study. Second, the P1p, N2po, and LPC TF scores were submitted to scalp PCAs (sPCA), which decomposed them into two scalp factors (SFs) for P1p and N2po and three for the LPC component (only those finally yielding significant effects are shown)

Figure 4 shows a selection of grand averages after subtracting the baseline activity (200 ms prestimulus recording) from each ERP. These grand averages correspond to the parieto-occipital channels, where significant results were expected. As indicated in the Method section, the first step was detecting and quantifying the relevant ERP components in the temporal domain through a tPCA. Consequently, thirteen temporal factors (TFs) were extracted and submitted to promax rotation. Three of them correspond to the components of interest based on their factor peak latency: TF7 (~ 140 ms), TF8 (~ 225 ms), and TF5 (~ 280 ms), associated with P1p, P2a, and N2x, respectively. We also decided to analyze the Late Positive Complex (LPC), a component sensitive to endogenous (controlled or top-down attention) rather than to exogenous attention (Chong et al. 2008; López-Martín et al. 2013) once some preliminary results were analyzed. The reason for this was that we suspected that the length of the stimuli presentation (800 ms, as indicated) was long enough to trigger this type of attention towards distractors after exogenous attention was developed. In other words, analyzing LPC allowed us to explore the exogenous-endogenous attention transition. The temporal factor associated with LPC was TF12 (~ 520 ms): see Fig. 5.

The second step was applying an sPCA to each temporal component to disentangle their spatial distribution and specifically select those spatial factors of interest. P1, P2, and N2 were decomposed into two spatial factors (SFs) each. As indicated in the Introduction, the spatial factors corresponding to the posterior P1 (P1p) and anterior P2 (P2a) topography, those related to exogenous attention, were selected for further analyses. In the case of N2, both spatial factors were selected since this family of components shows variable scalp distributions in studies of exogenous attention. Regarding the LPC component, which was decomposed in three SFs by sPCA, we selected the posterior spatial factor since it typically presents a parietal distribution in attention to emotional stimuli studies. As indicated in the Method section, the interaction of Motion x Emotion was explored only in those components previously shown to be sensitive to any of the main effects.

#### Control Analyses

As mentioned in the Method section, we carried out an ANOVA introducing Motion (dynamic, static) and Emotion (threatening, positive, and neutral) as factors on hEOG data to test any significant influence of horizontal ocular activity on potential experimental effects of components of ERP indexing exogenous attention (P1p, P2a, N2x). No significant differences were observed in hEOG at the latency range of these ERP effects (from 100 to 300 ms) [Emotion: F(2,140) = 2.925, *p* = 0.057, η^2^_p_ = 0.040; Motion: F(1,70) = 1.386, *p* = 0.243, η^2^_p_ = 0.019; Interaction F(2,140) = 0.085, G-G (0.069), corrected *p* = 0.907, η^2^_p_ = 0.001].

#### Experimental Effects on ERP Components

Table 4 shows the mean and standard deviation of all SF scores (equivalent to amplitudes, as indicated) corresponding to P1p, P2a, N2po, and LPC in each experimental condition. These factor scores were submitted to repeated-measures ANOVAs introducing Motion (dynamic – d–, static – s–) and Emotion (threatening - Neg-, positive - Pos-, neutral - Neu) as factors. The main outputs of these ANOVAs are also shown in Table 4.

**Table 4:** Means and standard deviations (in parenthesis) of P1p, P2p, N2a, N2po, and LPC of SF scores (linearly related with amplitudes) to each experimental condition and Results of the Two-way ANOVA with Emotion and Movement as factors (F, Probability [p], and effect size [η^2^_p_])

	Means (SD)						Emotion			Motion			Emotion x Motion		
	dNeg	sNeg	dPos	sPos	dNeu	sNeu	F (2,140)	*p*	η^2^_p_	F (1,70)	*p*	η^2^_p_	F (2,140)	*p*	η^2^_p_
TF7 (P1)															
TF7SF2 (posterior)	0.296 (0.895)	− 0.394 (1.260)	0.071 (0.934)	0.090 (0.945)	− 0.051 (0.802)	− 0.012 (1.004)	1.886	0.159	0.026	**8.923**	**0.004**	**0.113**	**17.237**	** < 0.001**	**0.198**
TF8 (P2)															
TF8SF1 (anterior)	− 0.023 (1.021)	− 0.076 (1.033)	− 0.108 (0.958)	− 0.012 (1.076)	0.233 (0.924)	− 0.038 (0.979)	2.055	0.134	0.029	1.318	0.255	0.018	3.929	0.022	0.053
TF5 (N2)															
TF5SF1 (anterior)	0.117 (0.870)	− 0.141 (1.026)	0.087 (1.004)	− 0.315 (1.047)	0.365 (0.915)	− 0.113 (1.014)	**5.162**	**0.007**	**0.069**	**37.310**	** < 0.001**	**0.348**	1.383	0.254	0.019
TF5SF2 (posterior)	− 0.118 (0.933)	0.021 (0.9969)	− 0.281 (0.912)	0.179 (1.135)	− 0.074 (0.887)	0.274 (1.044)	**3.510**	**0.033**	**0.048**	**28.158**	** < 0.001**	**0.287**	**3.771**	**0.025**	**0.051**
TF12 (LPC)															
TF12SF2 (posterior)	0.237 (0.954)	0.253 (0.905)	− 0.135 (1.038)	− 0.507 (1.038)	0.057 (1.004)	0.095 (0.981)	**24.739**	** < 0.001**	**0.261**	2.423	0.124	0.033	**4.575**	**0.013**	**0.061**

Significant and relevant results are shown in bold

*P1p (TF7; peak at 140 ms).* Results on the posterior SF (SF2), with an occipital distribution (Fig. 4), revealed a significant main effect Motion [*F* (1,70) = 8.923, *p* = 0.004, η^2^_p_ = 0.113], P1p amplitudes being greater for dynamic than for static stimuli. The interaction between Emotion and Motion also reached significance [*F* (2,140) = 17.237, G-G (0.979), corrected *p* < 0.001, η^2^_p_ = 0.198], and post-hoc comparisons showed the following results: i) within emotional conditions, dPos evoked greater amplitudes than sPos (*p* < 0.001), ii) within dynamic conditions, dPos evoked greater amplitudes than dNeu (*p* < 0.001), and iii) in static conditions, sNeg and sNeu trials elicited greater amplitudes than sPos (both *ps* < 0.001). The rest of the relevant pairwise contrasts were non-significant (Table 4).

*P2a (TF8; peak at 225 ms).* Results on anterior SF (SF1) did not reveal any significant main effect of Emotion [*F* (2,140) = 2.055, G-G(0.953), corrected *p* = 0.134, η^2^_p_ = 0.029] or Motion [*F* (1,70) = 1.318, *p* = 0.255, η^2^_p_ = 0.018]. Consequently, and as indicated, this component was discarded for further analyses.

*N2a (TF5; peak at 280 ms)*. Results on anterior SF (SF1), with an anterior distribution, revealed a significant main effect of Emotion [*F* (2,140) = 5.162, *p* = 0.007, η^2^_p_ = 0.069], N2a amplitudes being greater for Pos than for Neu (*p* = 0.001), the rest of pairwise comparisons being non-significant (all *ps* > 0.238). The main effect of Motion also reached significance [*F* (1,70) = 37.310, *p* < 0.001, η^2^_p_ = 0.348], with greater amplitudes for static relative to dynamic conditions (*p* < 0.001). The interaction of Emotion and Motion was not significant (*p* = 0.254).

*N2po (TF5; peak at 280 ms)*. ANOVAs revealed significant effects in SF2, which presented a parieto-occipital distribution (Fig. 4); hence, this component will be labeled N2po hereafter. These analyses revealed a significant main effect of Emotion [*F* (2,140) = 3.510, *p* = 0.033, η^2^_p_ = 0.048). Also in this case, post-hoc tests revealed a Pos condition elicited greater amplitudes (more negative) than Neu (*p* = 0.023), the rest of pairwise comparisons being non-significant (*p*s > 0.082). The main effect of Motion also reached significance [*F* (1,70) = 28.158, *p* < 0.001, η^2^_p_ = 0.287], N2po amplitudes being greater for dynamic than for static stimuli (*p* < 0.001). This component was also sensitive to the interaction of Emotion and Motion [F (2,140) = 3.771, *p* = 0.025, η^2^_p_ = 0.051]. Post-hoc comparisons yielded two significant pairwise contrasts: i) within dynamic conditions, dPos trials evoked greater amplitudes than dNeu (*p* = 0.003), ii) within static conditions, sNeg stimuli elicited greater amplitudes than sNeu (*p* = 0.020). The rest of relevant pairwise contrasts were not significant (Table 4).

*LPC (TF12; peak at 520 ms)*. The relevant SF (SF2), with a posterior distribution (Fig. 4), showed a main effect of Emotion [*F*(2,140) = 24.739, G-G (0.978), corrected *p* < 0.001, η^2^_p_ = 0.261]. Specifically, Bonferroni pairwise test indicated that amplitudes associated with Neg and Neu distractors presented greater amplitudes than those of Pos (both *p*s < 0.001). The interaction between Emotion and Motion also reached significance [*F*(2,140) = 4.575, G-G (0.976), corrected *p* = 0.013, η^2^_p_ = 0.061]. Post-hoc comparisons revealed three significant pairwise contrasts: i) within emotional conditions, dPos evoked greater amplitudes than sPos (*p* = 0.002), ii) within dynamic conditions, LPC amplitudes were greater for dNeg than for dPos (*p* = 0.012), and iii) within static conditions, sNeg and sNeu trials evoked greater amplitudes than sPos (*p* < 0.001 in both cases). The rest of relevant pairwise contrasts were non-significant (Table 4).

## Discussion

The main scope of this study was to explore exogenous attention to static and looming emotional distractors (3D-recreated). To this end, we employed a CDTD task consisting of a perceptual task and threatening, positive, and neutral animals that could remain static or looming toward participants as distractors. Based on previous research, we expected enhanced amplitudes to ERP components associated with exogenous attention (P1p, P2a, or N2x) and worse performance in the task (higher reaction times and/or error rates) when looming emotional and, more specifically, looming threatening distractors were presented. In general lines, we observed a sort of two-phase effect. Initially (before 300 ms), looming positive distractors captured exogenous attention to the greatest extent. Afterward, both static and dynamic negative distractors were associated with enhanced attention, as revealed by both behavioral and late ERP components. Importantly, and as discussed below, the first phase would reflect exogenous attention, whereas the second would be linked to endogenous attention.

The first phase was reflected in P1p and N2po components. Firstly, P1p, peaking at 140 ms, showed the opposite pattern in response to positive stimuli within dynamic and static conditions: maximal and minimal amplitudes, respectively. Negative and neutral stimuli did not elicit different amplitudes under their static and dynamic versions. This component is an early sensory component that constitutes an index of mobilization of automatic attentional resources (see a review in Hopfinger & Mangun 2001). P1p results indicate that motion adds relevance to positive valence by favoring attentional capture and point to this component as the earliest evidence of interaction between motion and emotion in exogenous attention. The scarce use of positive stimuli in previous studies exploring this interaction hinders us from comparing our results to previous ones, but we hypothesize that this result may be generalizable to other types of dynamic/static stimuli, which may be due to the positivity offset. This bias towards pleasant stimuli in a context of low-level arousal (Cacioppo et al. 1999) has an important survival value, enabling an organism in a neutral context to approach novel objects or stimuli and, ultimately, fostering the exploratory behavior. Since the neural activity underlying P1p reflects the processing of initial frames of each clip, this early positivity offset appears when stimuli are still relatively distant from participants (as indicated, distances were perceived as real thanks to the use of 3D/stereoscopic stimulation).

Later, the N2 component (peaking at 280 ms) showed experimental effects both in its anterior (N2a) and posterior (N2po) variants. Previous literature shows that, indeed, both anterior (Carretié et al. 2004; Feng et al. 2012; López-Martín et al. 2013) and posterior N2 components (Buodo et al. 2010; Carboni et al. 2017; Carretié et al. 2013, 2017; Eimer and Kiss 2007; Kosonogov et al. 2019) increase their amplitude in exogenous attention tasks when distractors are emotionally loaded. In our case, the emotional effect was corroborated for both components, showing increased amplitudes for positive stimuli and probably evidencing the positivity offset discussed above. Importantly, only the parieto-occipital N2 (N2po) component was sensitive to motion and the interaction effects of motion and emotion. Indeed, posterior N2 has been described as motion-sensitive independently of the direction of motion (Amenedo et al. 2007; Bach and Ullrich 1997; Hoffmann et al. 2001; Lorenzo-López et al. 2004; Pazo-Álvarez et al. 2004a). Regarding interaction effects, N2po amplitude was maximal in response to positive dynamic distractors and negative static ones. As in the case of P1p, the former interaction may also be reflecting the positivity offset mentioned above since processes underlying N2po (peaking at 280 ms but starting earlier) correspond to clip frames or events in which stimuli are closer than in the case of P1p, but relatively distant yet (closest positions take place at 800 ms). The latter interaction reveals that, in the static modality, threatening distractors were those showing maximal amplitudes. As explained below, this may reflect the first signs of the negativity bias, which will manifest more intensely in later phases. This bias favors the processing of aversive stimulation in situations of increased arousal (Cacioppo et al. 1999). The question arises on whether negative dynamic stimuli were not those evoking maximal N2po amplitudes (i.e., also eliciting a negativity bias). A probable explanation is that static stimuli, which consisted of the intermediate frame of each clip (i.e., the frame corresponding to the 400 ms of the 800 ms-length clip), were much closer than dynamic stimuli at the time they elicited N2po. Convergently, N2pc has been previously reported to increase in response to fearful faces presented within the peripersonal distance (as compared to farther distances: Martin et al., 2021). In this vein, evidence of preferential processing for objects located in near space, both behavioral (faster reaction times) and neural (earlier latencies and higher amplitudes in the N1 component), has been reported suggesting an attentional prioritization of stimuli appearing within reachable space. Additionally, fMRI data revealed that threatening stimuli presented close to the participants enhance brain activity in fear-relevant areas compared to stimuli presented further away (Coker-Appiah et al. 2013; Mobbs et al. 2010).

The last chronological phase was reflected both in LPC (520 ms) and behavior (798 ms average) and appeared to reflect the involvement of endogenous attention. Indeed, exogenous attention capture by a stimulus often leads to enhanced endogenous attention towards such stimulus if it remains in the visual scene (Corbetta and Shulman 2002; Theeuwes 2010, 1992). In our case, stimuli were displayed on the screen for 800 ms, a relatively long exposure as compared to other CDTD tasks (in which exposures usually range from 200 to 500 ms: Carretié et al. 2009, 2012; Erthal et al. 2005; Pessoa et al. 2005). This long exposure, forced by the characteristics of the clips, allowed the start-up of endogenous attention to distractors after exogenous attention had taken place. Endogenous attention has been reported to increase towards stimuli presenting emotional load as measured through behavioral and neural indices. Regarding the latter, the LPC component has been linked to endogenous rather than exogenous attention (e.g., see the reviews by Carretié 2014; MacNamara et al. 2012 and later works by Carboni et al. 2017; López-Martín et al. 2013). Indeed, the emotional content of pictures has been reported to enhance the amplitude of this component (for a review, see Olofsson et al. 2008, and later works by Feng et al. 2014; Fernández‐Folgueiras et al. 2021; Nordström and Wiens 2012). In our study, the LPC showed maximal amplitudes to dynamic negative distractors, but also static negative, in both cases differing from positive (but not from neutral, in the latter case). LPC reveals that once the threat reaches the peripersonal space (when neural processes eliciting this component occur, negative stimuli are perceived as very close through the 3D glasses), attention is significantly engaged. In this regard, LPC amplitudes have been shown to be sensitive to the proximity of stimulation, showing greater amplitudes for closer than for farther stimuli (Valdés-Conroy et al. 2014). Behavioral data have also shown improved task performance (lower reaction times and/or errors) when emotional content is endogenously attended (Del Zotto and Pegna 2015; Fernández‐Folgueiras et al. 2021; Yuan et al. 2014). Our results corroborate previous literature as threatening stimuli were associated with the highest accuracy in the line orientation task. These behavioral outcomes constitute the final single output of diverse neural discrete processes and, in our case, seem mainly determined by the final, endogenous, attentional effects.

Present results confirm that motion and emotion are mutually reinforced as preferential capturers of exogenous attention and that this interaction depends on the emotional valence and the distance of the stimulus at each moment. Thus, while positive dynamic stimuli capture attention to a greater extent in far distances, negative stimuli in the peripersonal space, both static and dynamic, attract exogenous (and finally endogenous) levels of attention. To our knowledge, this is the first study exploring exogenous attention to dynamic emotional stimuli using 3D technology, which provides greater ecological validity by ensuring a more realistic perception of looming motion. Although some low-level properties were balanced in our study (luminance, size, color), some others were not fully controlled; therefore, further physical characteristics should be considered in this respect in future studies on this topic. For example, controlling the motion of stimuli by homogenizing their dynamic pattern (smooth, abrupt, etc.) or employing more species and more exemplars within each species to reduce habituation processes are issues to consider in future studies. Furthermore, extending the subjective emotional assessment to dynamic stimuli, not only to static, would also be of interest. Finally, and in order to disentangle the specific effects of looming from the general effects of motion, at least another trajectory would be a necessary future step in this line of research. In any case, using a 3D presentation of animals seems an idoneous approach since they are associated with different emotional valences and may present both dynamic and static states in real situations.

## Data Availability

Data reduction preserving most of the variance resulted necessarily in further analyzing our Emotion (three levels: Pos, Neg, and Neu) × Motion Manipulation (two levels: Static or Dynamic), also taking into account that recordings consisted of a 59 EEG channels (+ vertical and horizontal EOG) × 505 digitized voltages or “time points” × 6 conditions matrix (this matrix is openly available at https://osf.io/5p94k/).
